# Single-photon detection in few-layer NbSe_2_ superconducting nanowires

**DOI:** 10.1038/s41467-026-75646-w

**Published:** 2026-07-29

**Authors:** Lucio Zugliani, Alessandro Palermo, Bianca Scaparra, Aniket Patra, Fabian Wietschorke, Pietro Metuh, Athanasios Paralikis, Domenico De Fazio, Christoph Kastl, Rasmus Flaschmann, Battulga Munkhbat, Kai Müller, Jonathan J. Finley, Matteo Barbone

**Affiliations:** 1 Walter Schottky Institute, TUM School of Computation, Information and Technology, and MCQST, Technical University of Munich, Munich, Germany; 2 Munich Quantum Instruments GmbH, Munich, Germany; 3 Department of Electrical and Photonics Engineering, Technical University of Denmark, Lyngby, Denmark; 4 Department of Molecular Sciences and Nanosystems, Ca’ Foscari University of Venice, Venice, Italy; 5 Walter Schottky Institute, TUM School of Natural Sciences, and MCQST, Technical University of Munich, Munich, Germany; 6 Present Address: Center for Sensors and Devices, Fondazione Bruno Kessler, Trento, Italy

**Keywords:** Superconducting devices, Two-dimensional materials, Single photons and quantum effects, Electronic devices, Optoelectronic devices and components

## Abstract

Superconducting Nanowire Single-Photon Detectors (SNSPDs) are key building blocks for photonic quantum technologies due to their ability to detect single photons with ultra-high efficiency, low dark counts and fast temporal resolution. Superconducting materials exhibiting high uniformity, large absorption cross-section and atomic-scale thickness are desirable to extend single-photon detection from the near-infrared up to the terahertz regime, where existing material choices are especially constrained. Substrate independence would further open the way to integrating detectors onto functional materials and heterostructures, enhancing performance and enabling proximal readout of a wide range of individual excitations. Here, we pattern the prototypical two-dimensional superconductor niobium diselenide (NbSe_2_) into few-layer nanowires less than 100 nm wide and demonstrate single-photon detection at 780 nm and 1550 nm. At the same time, the dark-count rate remains below 1 Hz up to the switching current and we achieve a timing jitter below 50 ps. We estimate via a diffusive hot-spot model that materials and geometric parameters would allow a theoretical cut-off wavelength in the millimetre range, assuming state-of-the-art device parameters. Our results open up routes toward quantum-limited detectors integrated into quantum-photonic circuits and quantum devices, with the potential for novel detection capabilities and unprecedented energy sensitivity.

## Abstract (web summary)

Demonstrating single photon detection with atomically thin superconductors remains a key challenge for scalable quantum photonics. Here, the authors report single-photon detection in few-layer NbSe_2_ nanowires, verified by linear count rate scaling and Poissonian photon statistics.

## Introduction

SNSPDs combine unmatched high-end performance across all key single-photon detection indicators^1,2^—from >99% detector efficiency (DE)^3^, to <5 ps timing jitter^4^, <10 ns recovery time^5^, and <1 Hz dark-count rates (DCR)^6^. Due to such performance metrics, SNSPDs have recently found many near-ideal applications in metrology, sensing and astronomy, and have cemented themselves as an indispensable ally in optical quantum computing and quantum communications^7,8^.

Development of material platforms has been at centre stage for quantum technologies^9^, and SNSPDs are no exception. Niobium-based superconductors (NbN, NbTiN) provide good timing resolution and fast recovery^10,11^, while amorphous silicides (MoSi, WSi) offer enhanced sensitivity to longer wavelengths^12–15^. Most recently, layered materials have emerged as a promising platform for quantum photonic technologies^8^, with potential applications spanning from deterministic arrays of single-photon emitters^16^, to quantum light emitting diodes^17^, to novel spin-photon interfaces for qubits^18,19^, and analogue quantum simulators^20,21^.

Layered materials offer a unique combination of advantages for single-photon detection, such as controllable atomic thickness (layer-by-layer) and fast thermalization time^22^ necessary for detecting low-energy excitations^23,24^; substrate independence, which allows for greater flexibility in device design, integration, and scope; as well as near-ideal uniformity due to sharp interfaces between layers, chemically terminated surfaces, and multi-*μ*m-scale single-crystal size, forecasting operational currents extremely close to the depairing currents as well as fast time resolution^25^. Such advantages are especially appealing for high sensitivity, fast detection of long-wavelength photons up to the terahertz range^26,27^, where available technologies remain limited^6^. Additionally, layered materials allow for the development of detectors integrated on-chip capable of sensing other degrees of freedom ranging from magnetic (spin) excitations to excitons, plasmons, phonons, and possibly even more exotic phases^28^.

The technological barriers to using layered materials for single-photon detection^29^ were only recently overcome in the high-temperature layered cuprate superconductor Bi_2_Sr_2_CaCu_2_O_(8−*δ*)_^30^ and in moiré superconductor magic-angle twisted bilayer graphene (MATBG)^31^, showcasing the potential of layered materials for single-photon counting devices. However, no SNSPDs with conventional layered superconducting materials, which promise to offer a superior combination of near-atomic thickness (single layer), scalability, speed, and sensitivity to low energies, have yet been reported. Niobium diselenide (NbSe_2_) is a van der Waals material with a temperature-dependent phase diagram including a normal metallic phase, hosting charge density waves^32^. For temperatures below 10 K, it exhibits a layer-dependent superconducting transition that persists down to the three-atom, ~0.7 nm-thick monolayer limit^33,34^. NbSe_2_ offers high absorption, ~1 ps carrier thermalization^22^ and can be processed using standard top-down nanofabrication techniques^35^. Moreover, it can be grown using scalable methods, such as vapour deposition at the wafer scale^36^, and benefits from integration with other van der Waals materials, such as hexagonal boron nitride (hBN). Few-layer NbSe_2_ was used for multi-photon detection in the NIR^29^ and terahertz^37^, and revealed hints of discrete-photon sensitivity when patterned into nanowires^38^, but detecting single photons remained elusive until now. Here, we report the linear increase of the photon count rate under varying photon excitation power in a 7-layer-thick top-hBN encapsulated NbSe_2_ superconducting nanowire. The count rate follows a Poisson distribution for single events under coherent (laser) excitation at 780 nm and 1550 nm with an incident power spanning multiple orders of magnitude, demonstrating single-photon detection^1,39^.

## Results

### Device fabrication and electrical behaviour

**Fig. 1:**
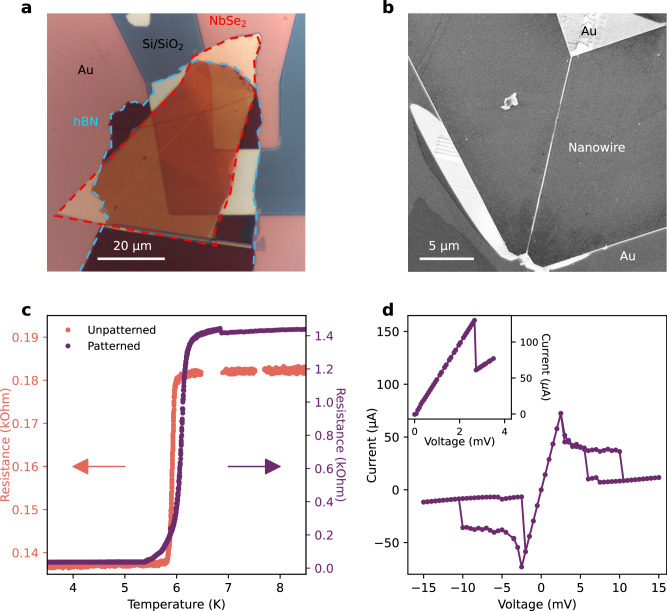
Fabrication and electrical behaviour at cryogenic temperature of NbSe_2_-nanowire devices. **a** Optical image of a hBN/NbSe_2_-heterostructure transferred onto pre-patterned contacts (pink). **b** SEM image of the device after shaping the transport channel into a ~100 nm nanowire. **c** Resistance as a function of temperature shows a superconducting transition of 6 K for a seven-layer crystal before and after patterning. **d** *I*-*V* characteristics at 1 K of the patterned device: in the positive regime, the switching current at 2.4 mV is followed by an intermediate current state where superconductivity is broken and recovered up to 10 mV, where the wire becomes fully resistive. The inset shows the *I*-*V* characteristics becoming a single, sharp transition from superconducting to resistive by adding a shunt resistor.

Figure 1 summarizes the device fabrication flow. We assemble stacks of multilayer NbSe_2_/hBN on oxidized silicon wafers according to conventional micromechanical cleavage, followed by dry-transfer processes^40,41^. The stacks are transferred on top of pre-patterned Ti/Au electrodes, with the NbSe_2_ layer in contact with the metal and the oxidized silicon substrate (Fig. 1a). Subsequently, we shape the heterostructures into 60–100 nm wide nanowires using standard nanofabrication techniques (Fig. 1b). For our proof-of-concept devices, we chose the simplest geometry providing functionality without straining fabrication overhead. Further details on the fabrication process are included in the Methods Section.

We then study the two-point electrical transport behaviour of the devices at cryogenic temperatures (see Methods for details on the experimental setup). Figure 1c shows typical superconducting transition temperature measurements of a selected device pre- and post-patterning of the nanowire (see Supplementary Information for transition temperatures of nanowires with different thicknesses, Supplementary Fig. 2). The critical temperature for both the unpatterned and patterned device is ~6.0 K and the superconducting transition occurs sharply being ~0.5 K broad, which confirms the homogeneity of the flake and is in good agreement with previously reported values for both unpatterned^33,34^ and patterned^38^ few-layer (≤10) NbSe_2_ flakes. After patterning the heterostructure to form a nanowire, we observe a change in the NbSe_2_ film resistance given the narrower geometry of the patterned device, increasing to 1.40 kΩ, translating to a resistivity *ρ* ~ 6 μΩ ⋅ cm (sheet resistance *R*_□_ ~ 14 Ω/□). These values are more than an order of magnitude lower than conventional sputtered superconducting films^4^ due to the homogeneity of the crystal. In contrast, the critical temperature remains minimally affected by top-down patterning. The residual resistance of ~100 Ω below the superconducting transition is due to the contact resistance between NbSe_2_ and the metal contacts as well as the wiring integrated in the setup.

Figure 1d shows the *I*-*V* characteristics measured at ~1 K, which allows us to extract the switching current *I*_sw_ corresponding to the superconducting-to-resistive transition. Initially, the nanowire is in a superconducting state. While increasing the voltage, we see an ohmic increase of the current governed by the contact and the wiring resistance of the setup. At about 2.4 mV, the *I*-*V* curve reaches a regime where the device is in a state of intermediate resistance. This is caused by the oscillation between superconducting and normal conducting state occurring because of the short recovery time of the device related to its low kinetic inductance^42^. Increasing the bias voltage further, we reach a second ohmic regime where the detector switches fully to the normal conducting state. This behaviour is also observed for reverse voltage. To remove the intermediate oscillatory behaviour and obtain a single clear phase transition, we connect a resistor in parallel to the device. The inset shows the *I*-*V* characteristics recorded in such case. In this configuration, the current is redirected from the device to the resistor, decreasing Joule heating and therefore allowing a faster relaxation to the superconducting state. We note that in this case we observe a sharp transition between superconducting phase and normal-conducting states, due to the decoupling of the electro-thermal components of the device.

### Photoresponse characteristics

**Fig. 2:**
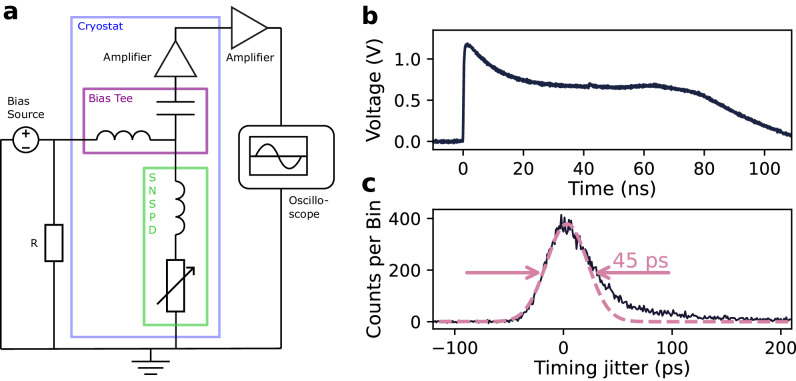
Photoresponse characteristics of the detectors. **a** Schematic of the electronic read-out circuit. A voltage source is used to supply a bias current to the detector. A parallel resistor *R* = 50 Ω is utilized to mitigate latching. The current is supplied to the detector via the DC arm of a bias-tee (purple) installed inside a cryostat working at 4 K (blue). The detector (green) is represented by an inductance *L*_*k*_ in series with a variable resistor. When a photon is absorbed, the sudden increase in resistance generates a voltage pulse travelling through the AC arm of the cryogenic bias-tee (purple) and is consecutively amplified both at cryogenic temperature as well as at room temperature. The signal is then recorded via an oscilloscope. **b** time-dependent voltage pulse. The decay initially follows conventional single-exponential behaviour but then shows latching, where the superconducting state is not recovered until the current is redirected through the parallel resistor. **c** Timing jitter of the device with the pulse shown in b, where the Gaussian fit returns a full-width-at-half-maximum value of 45 ps.

We measure the photoresponse of the detectors in a cryogenic setup at ~1 K, see Methods for details. Figure 2a shows a schematic of the read-out circuit. The source current flows through a bias-tee formed of a DC filter (capacitance) and an AC filter (inductor). From there, the current flows through the detector, which can be represented as a kinetic inductance *L*_*k*_ in series with a time-dependent resistance *R*_*n*_(*t*). Absorbed photons break the superconducting state, abruptly increasing the resistance, redirecting the current back to the bias tee and across the DC filter, to the signal output. The signal is amplified using both a cryogenic and a room-temperature amplifier. Finally, the signal is measured as a voltage pulse *V*(*t*) using an oscilloscope or a counter. Light is guided to the detector via optical fibre centred at 780 nm and 1550 nm. A pulsed laser (50 MHz repetition rate) is used to characterize the timing jitter of the device, where a correlation measurement is performed between the trigger signal generated by the laser and the detector response.

Figure 2b shows a photon detection pulse recorded by our setup as a time-dependent voltage response *V*(*t*). The voltage trace initially follows a conventional exponential decay; however, it does not fully relax to 0 V but remains at a finite voltage due to latching behaviour, whereupon the device reaches a metastable state in which the current-induced Joule self-heating exceeds the electron cooling process. Latching in NbSe_2_ is especially favoured by the low *R*_□_. Redirection of the current through the parallel resistor speeds up recovery of the superconducting state (see Supplementary Information for details and full-pulse analysis, Supplementary Fig. 3).

The temporal resolution of SNSPDs is strongly influenced by intrinsic material properties like low kinetic inductance, fast quasi-particle thermalization and crystallinity^4^. The pristine quality and the associated properties make NbSe_2_ a promising candidate for fabricating devices with exceptionally low timing jitter. To provide an initial assessment of the temporal characteristics offered by this novel platform, we measure the timing jitter, or the time distribution of output signals upon photon absorption. Figure 2c shows the histogram of the detection events recorded by the time tagger as a function of time. By fitting the data with a Gaussian distribution, we report a full-width at half maximum of ~45 ps, which sits already within the range of commercially available SNSPD systems, and only an order of magnitude away from the highest temporal resolution ever reported^4^, achieved with devices optimized to simultaneously minimize the longitudinal geometric and intrinsic jitter.

### Single-photon detection

**Fig. 3:**
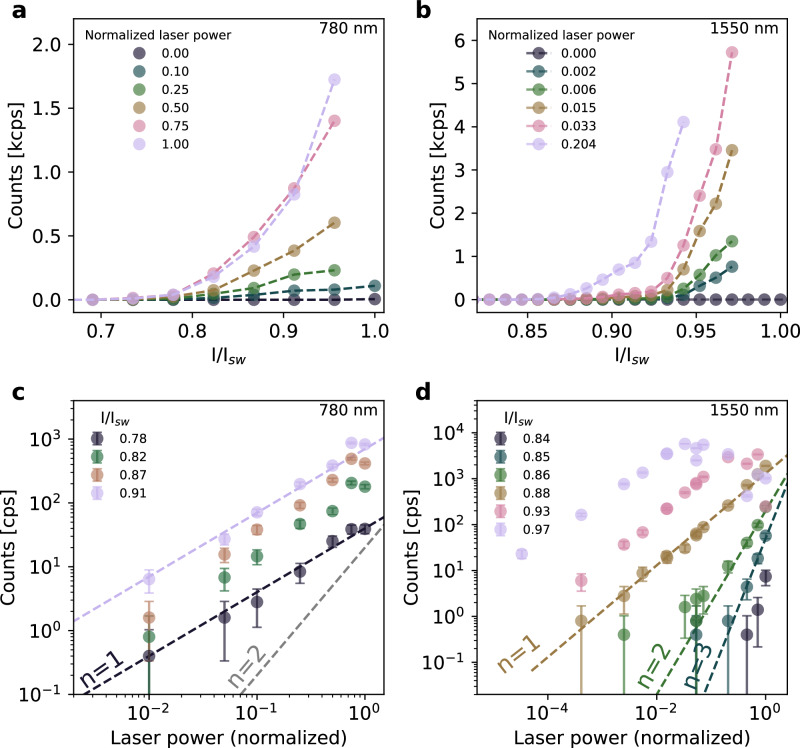
Single-photon detection in a superconducting NbSe_2_ nanowire. Count rates as a function of normalized current *I*/*I*_sw_ under increasing laser power at **a** 780 nm and **b** 1550 nm, with counts increasing exponentially until reaching *I*_sw_, after which the device turns into the resistive state, indicating sub-unity internal detection efficiency (error bars are shot-noise-limited and are smaller than the marker size). Count rates as a function of laser power for several currents at **c** 780 nm and **d** 1550 nm, where the uncertainty is shot noise limited. Dashed curves indicate power-law behaviour with variable integer exponent *n* = 1–3. The count rates perfectly follow the dashed curves *n* = 1 over multiple orders of magnitude, indicating single-photon detection.

We continue to focus our attention on the photo-detection behaviour when illuminating the nanowires with CW laser light at 780 nm and 1550 nm. Figure 3a, b show the count rates in the same nanowire device as a function of applied voltage. Below a normalized current threshold, normalized to the switching current, *I*/*I*_sw_ ~ 0.75 (*I*/*I*_sw_ ~ 0.85) at 780 nm (1550 nm), the detector does not record counts. Once we cross the minimum current threshold, the device becomes sensitive, showing exponentially increasing counts for increasing current, as expected, up to >0.95 *I*/*I*_sw_ (0.97 *I*/*I*_sw_) at 780 nm (1550 nm), while maintaining the dark count rate <1 Hz. A detector of unitary internal efficiency would reach and maintain a saturation count rate until reaching the critical voltage turning the NbSe_2_ from superconducting into resistive. However, our data show a sharp fall from the maximum count rate to *I*_sw_ without a saturation plateau. The sub-unitary internal detection efficiency can be explained by the interplay between dead time—the bias current recovery time—and cool-down time—the resistive hotspot lifetime. For increasing count rates, the cool-down time increases compared to the dead time, making the device more prone to latching. This limits the count rate and decreases the maximal biasing current^42^. Modifying the geometry of the device—reducing its cross-section—and enhancing its transport properties—increasing the bias current closer to the depairing current—could avoid latching and therefore reach saturation.

To quantify the photon sensitivity, we recall that the probability distribution of detecting *n* photons from a coherent source when *m* photons are on average expected is described by the Poisson distribution $\wp=\exp (-m)({m}^{n}/n!)$^43^. If *m* is sufficiently small^1^, ⪅0.1, *℘* ≃ (*m*^*n*^/*n*!), which is a power-law function where *n* becomes easily identifiable as the slope on the log-log representation of the count rate versus average power. For single-photon detection events, by definition *n* = 1, which yields a linear increase of the count rate with increasing photon flux. Figure 3c, d show the detector count rates as a function of laser power at 780 nm and 1550 nm, respectively, for several applied biases, compared to curves of discrete power exponent *n* = 1, 2 and 3. At 780 nm, the count rates exhibit a clear linear increase across two orders of magnitude at all different values of *I*/*I*_sw_, confirming single-photon sensitivity. At 1550 nm, starting from the lowest applied biases, initially the count rates increase superlinearly with exponents matching first *n* = 3 and then *n* = 2. Beyond a current threshold *I*/*I*_sw_ ~ 0.88, the slope reduces to a linear increase over more than three orders of magnitude, unequivocally indicating counting of single-photon events.

### Perspectives for low-energy detection

**Fig. 4:**
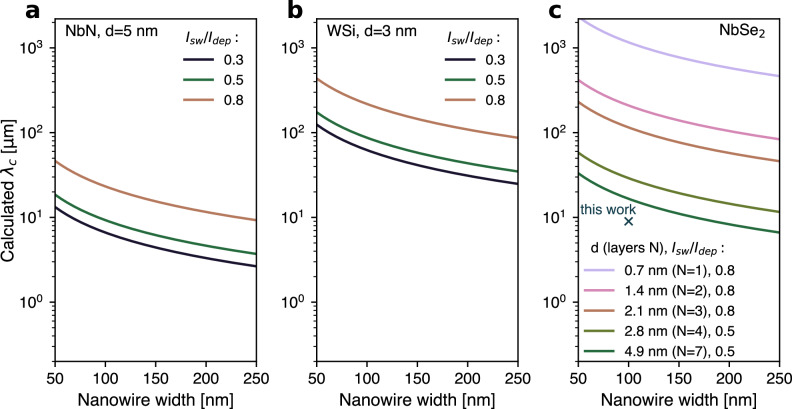
Predicted cutoff wavelength for representative SNSPD material platforms. **a** In first-generation NbN devices, theoretical *λ*_*c*_ is limited to a few tens of micrometres. **b** Second-generation amorphous WSi devices extend this value by about an order of magnitude, to beyond 100 μm. **c** NbSe_2_ nanowires of similar geometry are expected to increase *λ*_*c*_ by another order of magnitude, breaking the millimetre barrier. The cross represents the predicted cutoff wavelength for the device measured in this work, with a *I*_bias_/*I*_dep_ < 0.1.

Assuming that future research developments in NbSe_2_ SNSPDs can match the significant improvement of device parameters reached in technologically mature systems, we explore the advantages offered by NbSe_2_ material parameters for long-wavelength detection. To provide a perspective in this direction, we calculate the cutoff-wavelength *λ*_*c*_ of SNSPDs based on their material and device parameters using a simple diffusion-based hot-spot model approximation^23^, the detection mechanism taking place in the limits of low *D* and *τ*_th_ of NbSe_2_^23,44^:

1 $${\lambda }_{c}=\frac{hc\zeta }{{N}_{0}{\Delta }^{2}dw\sqrt{{{{\rm{\pi }}}}D{\tau }_{{{{\rm{th}}}}}}}{(1-{I}_{{{{\rm{bias}}}}}/{I}_{{{{\rm{dep}}}}})}^{-1}$$

with *ζ* the conversion efficiency, Δ the superconducting gap, *N*_0_ the density of states at Fermi energy, *D* the diffusivity, *τ*_th_ the thermalization time, *I*_bias_ and *I*_dep_ the bias and depairing (the theoretical critical limit) current, *w* the detector’s width, and *d* the detector’s thickness. NbSe_2_ offers especially favourable parameters: low *D*^38^, fast *τ*_th_^22^, and sub-nm thickness. In Fig. 4 we compare *λ*_*c*_ for three detectors made of different geometries and materials: a NbN, the workhorse of first-generation SNSPDs; WSi, currently the record-holder for long-wavelength detection^6^; and NbSe_2_ (see Supplementary Information for calculations’ details and parameters, Supplementary Tables I, II and III). Since the quasi-particle multiplication efficiency as well as the current component are strongly affected by technological maturity, underestimating the potential of newer material systems, we calculate *λ*_*c*_ assuming *ζ* = 1 and *I*_bias_/*I*_dep_ as a free parameter in a realistic range achievable by state-of-the-art devices^45,46^. Figure 4a shows that NbN is theoretically limited to detecting wavelengths up to a few tens of μm, as is indeed the case in practice despite *I*_bias_/*I*_dep_ well above 0.5^46^. Figure 4b shows that WSi allows about an order of magnitude improvement to >100 μm, thanks to lower density of states, lower critical temperature, and thinner achievable films^6^. These calculations compare reasonably well with the recent results brushing detection at 30 μm^6^ for a device with *I*_bias_/*I*_dep_ ~ 0.3. Impressively though, Fig. 4c shows that a two-layer NbSe_2_ matches the *λ*_*c*_ of WSi for the same *I*_bias_/*I*_dep_ and crosses 1 mm for a single-layer crystal with *I*_bias_/*I*_dep_ ~ 0.8, deep into the terahertz (0.03 to 3 mm) range. The cross represents the predicted cutoff wavelength for the device measured in this work, with a *I*_bias_/*I*_dep_ < 0.1.

## Discussion

Immediate next steps include optimizing detector fabrication, including geometries and architectures—such as meander-shape geometry or on-chip inductors and resistors—to minimize latching and maximise photon absorption at the operational wavelength. Moreover, an increased wire length would increase the device inductance and reduce the contact resistance to a negligible contribution to the detection pulse generation. With these implementations, single-photon detection can be expanded to the MIR (>2 μm).

On the longer run, substrate independence will allow for greater flexibility in device design, enabling seamless incorporation of layered materials’ SNSPDs into virtually any dimensionally compatible functional structure. Further, their nature and dimensionality will benefit from integration with a broader spectrum of conventional and quantum materials^47,48^ to enhance device functionality. Collectively, these features form a broad parameter space to explore for the realization of next-generation on-chip integrated quantum photonic circuits as well as detectors for free-space optical communication, astronomy, and chemical sensing from the near-IR up to the terahertz range. Finally, embedding atomically thin superconducting single-excitation detectors into layered materials heterostructures or similar quantum devices may offer a new tool towards local probing of proximal low-energy phases^49^, adding novel exploratory options for metrology and basic physics studies.

## Methods

### Sample preparation

Electrical contacts (Ti/Au 5/20 nm) were pre-patterned using optical lithography and electron-beam evaporation on Si/SiO_2_ substrates. NbSe_2_ and hBN flakes (from 2D Semiconductors) were mechanically exfoliated from bulk crystals on PDMS and Si/SiO_2_ substrates (SiO_2_ 70 nm thick), respectively. The flakes were selected based on their optical contrast, shape, and cleanliness. hBN crystals are about 20 nm thick, while NbSe_2_ crystals have a variable thickness from about 10 nm down to 2 layers. The devices were assembled via dry-transfer techniques: hBN was picked up from Si/SiO_2_ using polycarbonate films^41^, followed by NbSe_2_ picked up from PDMS on the same stamp^40^. Then, the entire heterostructure was released on the pre-patterned contacts. Heterostructures were then patterned by electron beam lithography, followed by reactive ion etching with an SF_6_+Ar gas mixture. The nanowire structure around the contact pads was designed to ensure a robust contact between the nanowire and the contact pads.

### Photoelectric setup

We perform cryogenic measurements in a closed-cycle Adiabatic Demagnetization Refrigerator (Kiutra L-type). Photoresponse measurements are performed at 1 K. Bias to the samples is applied with a voltage source *V*_*b*_ (Keithley 2450). Optionally, a resistor in series *R*_*b*_ is applied after the biasing source. The series resistor transforms the voltage bias into a current bias, *I*_bias_ = *V*_*b*_/*R*_*b*_, stabilizing the current in the circuit. A shunt resistor is applied in parallel, dissipating part of the current when the detector is in the normal conducting state, to facilitate the recovery of superconductivity. The current enters the cryogenic setup and flows through the bias-tee, which consists of a DC filter (capacitor) and an AC filter (inductor). From there, the current gets directed to the detector, acting as an inductance *L*_*k*_ (or kinetic inductance), in series with a time-dependent resistance *R*_*n*_(*t*), in an equivalent circuit diagram. Absorbed photons or noise abruptly increase resistance, redirecting the current back to the bias tee and across the AC filter. The signal is amplified using both a cryogenic and a room-temperature amplifier. Finally, the signal is measured as a voltage pulse *V*(*t*) using a counter or an oscilloscope. The sample can be illuminated with two fibre-coupled laser sources, at 780 nm and 1550 nm, whose light is directed into the cryostat to the sample through a fibre attenuator and a beam splitter for the power measurement. One of the two beams is guided to the cryostat, the other to a power metre. The light is collimated to illuminate the sample homogeneously (beam diameter  ~ 2 mm). To characterize the timing jitter of the device, we measure the time correlation between the trigger time of a pulsed laser at 1550 nm (repetition rate 50 MHz) and the SNSPD detection time.

## Supplementary information

Supplementary Information

Transparent Peer Review file

## Data Availability

The data generated and analyzed in this study have been deposited in the mediaTUM database accessible with the following link: 10.14459/2026mp1851786.
