# Supplementary material for: Single-photon detection in few-layer NbSe2 superconducting nanowires
Source: Nat Commun. 2026 Jul 29;17:7586. doi: 10.1038/s41467-026-75646-w (PMC13421474; doi:10.1038/s41467-026-75646-w)
Supplement: Supplementary file 1 — Supplementary Information [file 41467_2026_75646_MOESM1_ESM.pdf]

Supplementary Information to:
Single-Photon Detection in Few-Layer $NbSe_2$ Superconducting Nanowires

Lucio Zugliani,[1,*] Alessandro Palermo,[1] Bianca Scaparra,[1] Aniket Patra,[2] Fabian Wietschorke,[1] Pietro Metuh,[3] Athanasios Paralikis,[3] Domenico De Fazio,[4] Christoph Kastl,[5] Rasmus Flaschmann,[1] Battulga Munkhbat,[3] Kai Müller,[1] Jonathan J. Finley,[5,†] and Matteo Barbone[1,‡]

[1] *Walter Schottky Institute, TUM School of Computation, Information and Technology, and MCQST, Technical University of Munich, Munich, Germany*
[2] *Munich Quantum Instruments Gmbh, Munich, Germany*
[3] *Department of Electrical and Photonics Engineering, Technical University of Denmark, 2800 Kgs. Lyngby, Denmark*
[4] *Department of Molecular Sciences and Nanosystems, Ca' Foscari University of Venice, Venice, Italy*
[5] *Walter Schottky Institute, TUM School of Natural Sciences, and MCQST, Technical University of Munich, Munich, Germany*

(Dated: July 6, 2026)

SEM IMAGE OF FABRICATED DEVICE

Supplementary Figure 1 shows an SEM image of the fabricated device close to one of the contact pad area. The wire has a width ~100 nm and it is directly in contact with the SiO_2 substrate.

SUPERCONDUCTING TRANSITIONS OF PATTERNED DEVICES

In Supplementary Figure 2, the resistive transition for several patterned devices is shown. The values of resistance are normalized, removing the contact resistance, and are shifted for clarity. The transition temperatures varies based

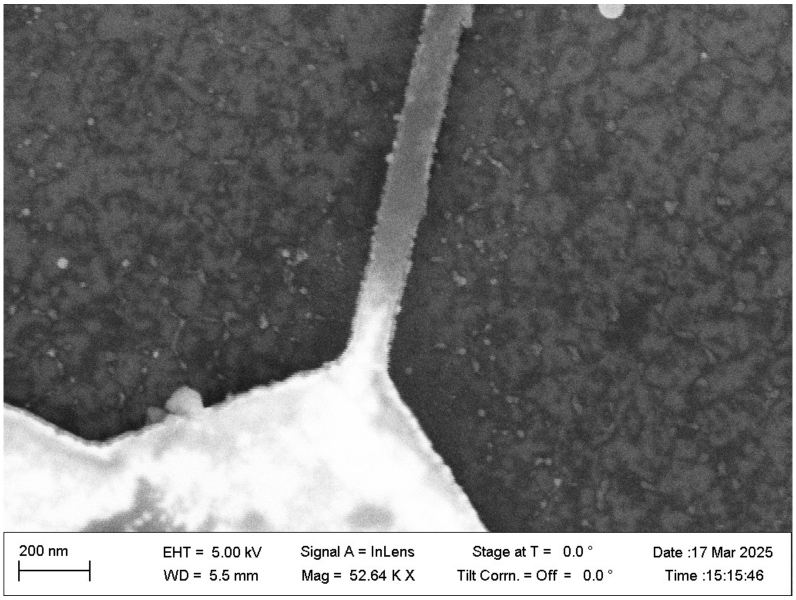

Supplementary Figure 1. SEM image of fabricated deivce.

* lucio.zugliani@tum.de
† jj.finley@tum.de
‡ mbarbone@fbk.eu; Current affiliation: Center for Sensors and Devices, Fondazione Bruno Kessler, Trento, Italy

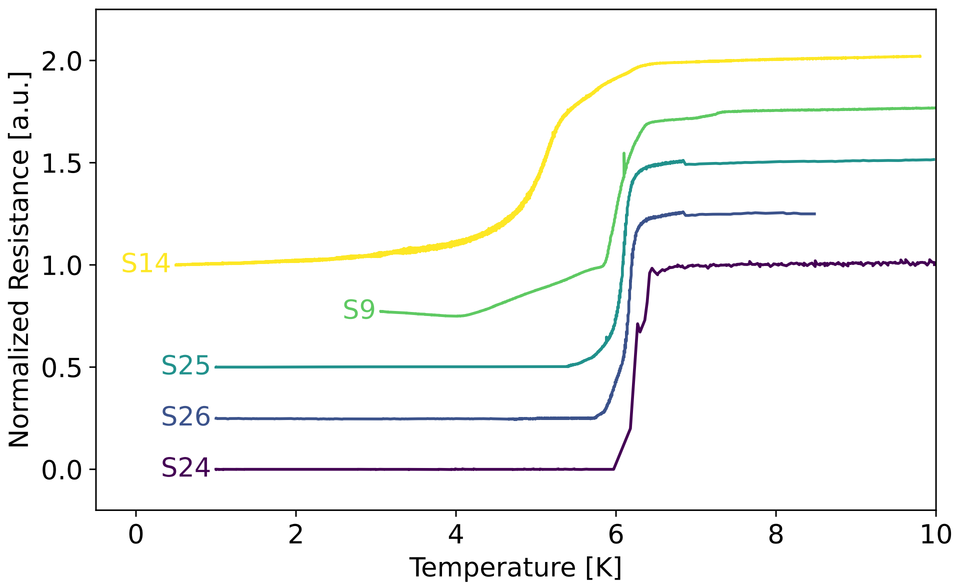

Supplementary Figure 2. Superconducting transitions of patterned devices.

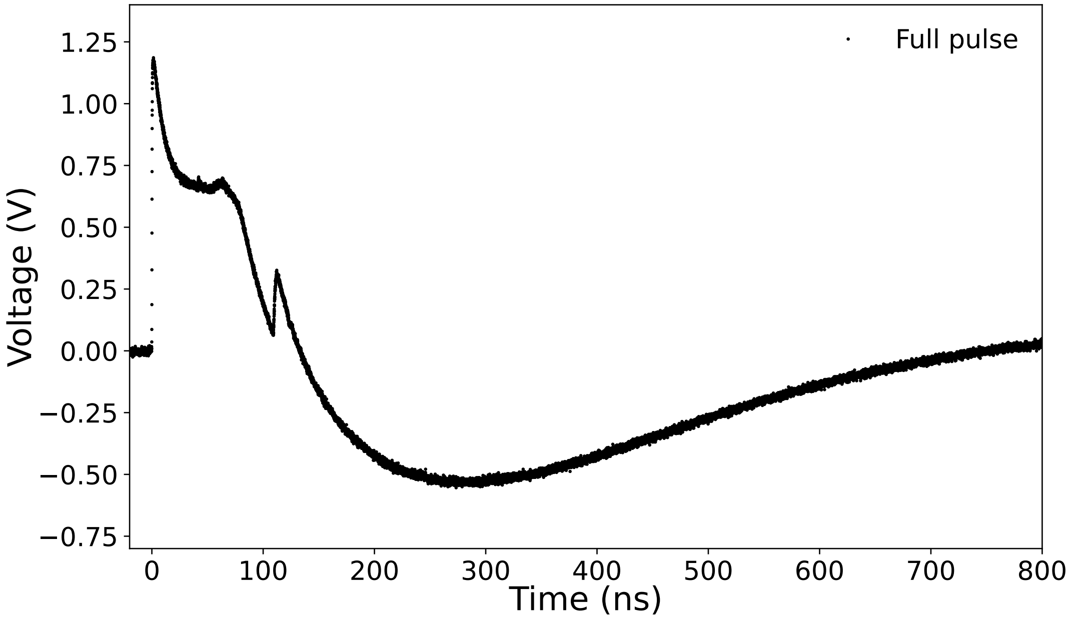

Supplementary Figure 3. Extended detection pulse.

on the number of $NbSe_2$ layers in the device, ranging between 5K and 6.5K. The samples presented have the following number of layers: S14 - 2 layers; S9 - 3 layers; S25 - 7 layers; S26 - 5 layers; S24 - bulk.

EXTENDED PULSE

The pulse initially decays exponentially before reaching the latching plateau from ∼20 ns to ∼100 ns. Around 110 ns a reflection from the wiring causes a spike (Supplementary Figure 3). After that, the pulse undershoots below 0 V

due to the instrumental effect of the cryogenic amplifier, until it fully recovers ∼800 ns.
The latching behaviour is mainly governed by the kinetic inductance of the device, which can be estimated by $L_k = \frac{\hbar R}{\pi \Delta} = \frac{\hbar R}{1.764 \pi k_B T_c}$ [1], where R is the resistance of the device above the superconducting transition, and T_c is the critical temperature. The device measured here has $L_k \approx 319$ pH. Given the low value of L_k, current is restored in the device before complete thermalization, thus before restoring the superconducting state, and therefore it results in a finite resistance. The superconducting state is then recovered (transition from non-zero voltage to 0) due to the implementation of the parallel resistor at room temperature. The fact that the current in the device is not immediately redirected to the parallel resistor comes from the circuit configuration implemented, given that the AC filter is in between the device and the shunt.

EFFECT OF LIGHT ILLUMINATION ON SWITCHING CURRENT

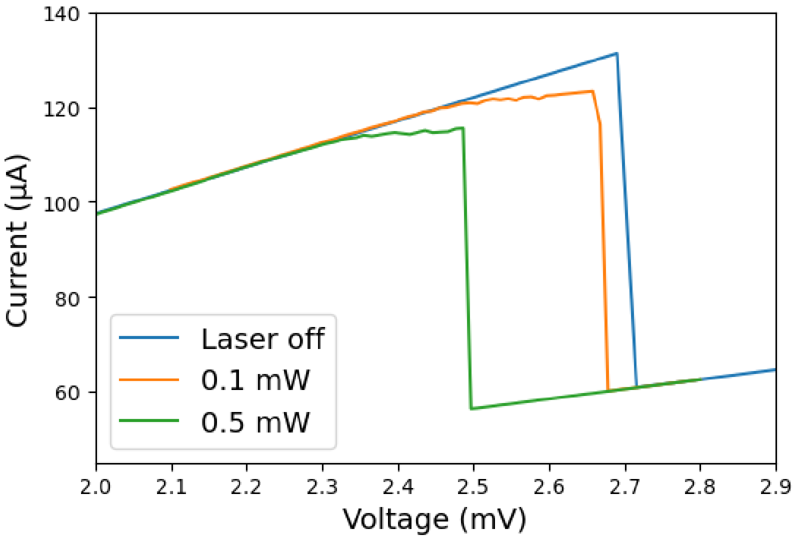

Supplementary Figure 4. **I-V curves under light illumination.**

The I-V curves for the device are measured under illumination with different 1550 nm laser powers (Supplementary Figure 4). We notice a change in the switching current depending on the applied laser power. Increasing the laser power, we register a decrease of I_{sw} - the sudden drop in current in the I-V sweep - as expected due to the higher photon flux.
During illumination, the I-V curve do not present a sharp transition to the normal conducting state. This can be explained by the latching state of the device, as explained in Section EXTENDED PULSE.

PULSE TRAIN

The time trace recorded with an oscilloscope is shown in Supplementary Figure 5. The time window has a length of 50 ms, where several pulses, as the one presented above are recorded.

DEPAIRING CURRENT CALCULATION

We calculate the estimated cutoff wavelength of a device measured in this work. To do so, we use the parameters presented in Supplementary Table I and II, which are measurement parameters (T), extracted from our device (nanowire width w, superconducting gap Δ, sheet resistance $R_\square$, critical temperature T_c) or taken from literature (diffusion coefficient D)[2]. The depairing current is calculated using the Bardeen formula [3]:

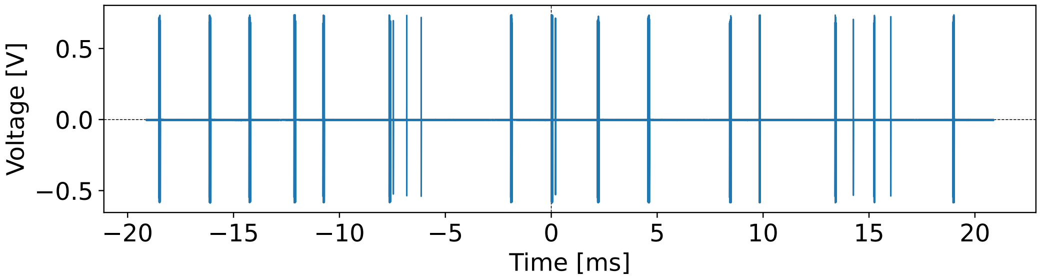

Supplementary Figure 5. **Detection pulses under illumination.**

$$I_{\text{dep}}^{\text{Bardeen}} = \frac{0.74\, w\, \Delta(0)^{3/2}}{R_{\Box}\sqrt{\hbar D}}$$

with $R_{\Box} = (2e^2 DdN(0))^{-1}$. With e electron charge, d thickness, $N(0)$ density of states. To account for finite Temperature, we multiply $I_{\text{dep}}^{\text{Bardeen}}$ by the following interpolation T_{dep} away from T_c, as is our case: $T_{\text{dep}} \sim \left[1-(T/T_C)^2\right]^{3/2}$ [4].

Supplementary Table I. Material and device parameters used to calculate depairing current.

**Parameter**	**Value**
w [nm]	100
$\Delta = k_B T_c$ [meV]	0.516
$R_{\Box}$ [$\Omega/\Box$]	14
D [$\mathrm{m^2s^{-1}}$]	1×10^{-5} [2]
T [K]	1
T_c [K]	6

CUTOFF WAVELENGTH EFFICIENCY CALCULATIONS

Supplementary Table II. Material parameters used in the diffusion-based hot-spot model.

**Parameter**	**$NbSe_2$**	**WSi**	**NbN**
N_0 [10^{47} $\mathrm{m^{-3}J^{-1}}$]	1.3[5]	1.08[6]	1.6[7]
$\Delta = k_B T_c$ [meV]	0.43 (T_c = 5 K)	0.112 (T_c = 1.3 K)	0.43 (T_c = 5 K)
D [$\mathrm{m^2s^{-1}}$]	1×10^{-5} [2]	5.3×10^{-5} [6]	3.5×10^{-5} [7]
τ_{th} [ps]	10[8]	120[9, 10]	12[10, 11]
d [nm]	0.7-bulk	3[6]	5[7]

To calculate the cutoff wavelength according to the diffusion-based hot-spot model described in the main text, we employ materials' parameters provided in Supplementary Table II. We adopt a ζ=1 for all materials to adopt a prudent approach, although due to lower carrier density, $NbSe_2$ is expected to have a higher ζ than conventional superconductors [14]. We calculate $N_0^{NbSe_2}$=$1.3{\times}10^{47} m^{-3} J{-}1$ from 2.7 states per unit cell[5] with cell volume V_{cell}=$(\sqrt{3}/2)a^2c$, a=3.45 $\mathring{A}$, c=12.550 $\mathring{A}$. We calculate Δ=$k_B T_c$, with k_B the Boltzmann constant, from the superconducting transition temperatures T_c^{WSi}=1.3 K[6], T_c^{NbN}=5 K [7], and $T_c^{NbSe_2}$ varies based on the number of layers in the device, following values from literature and from this work presented in Supplementary Table III[12, 13]. We calculate $D^{NbSe_2} = 1 \times 10^{-5}\ m^2 s^{-1}$ from magnetic field dependence data of the superconducting transition in a similar device [2]. Finally, τ_{th} is ultimately limited by the electron-phonon scattering time, which is the value we use, although the real thermalization time will be slightly different. In the case of crystalline NbN the value is ∼12 ps[11]. For few-nm-thick

Supplementary Table III. Thickness dependent critical temperature for $NbSe_2$.

Layers	Cao *et al.* [K] [12]	Xi *et al.* [K] [13]	This work [K]
1	1.95	3.1	-
2	4.5	5.4	5.1
3	5.5	5.9	5.6
4	-	6.2	-
7	6.5	6.9	6.0

amorphous WSi films, the amorphous nature of the material increases the electron-phonon scattering time to ∼120 ps [9, 10]. In $NbSe_2$, this value falls to a shorter ∼10 ps [8]. We take all values as close as possible to operation temperature.

To calculate the data point in Figure 4, we used the parameters presented in Supplementary Table I, which are measurement parameters (T), extracted from our device (w, Δ, $R_\square$, T_c) or taken from literature (D)[2].

[1] M. Tinkham, *Introduction to Superconductivity*, 2nd ed. (Dover Publications, 2004).
[2] P. Metuh, A. Paralikis, P. Wyborski, S. Jamo, A. Palermo, L. Zugliani, M. Barbone, K. Müller, N. Gregersen, S. Vaitiekėnas, J. Finley, and B. Munkhbat, ACS Photonics **12**, 5912 (2025).
[3] J. Bardeen, Reviews of Modern Physics **34**, 667 (1962).
[4] Y. P. Korneeva, D. Y. Vodolazov, A. V. Semenov, I. N. Florya, N. Simonov, E. Baeva, A. A. Korneev, G. N. Goltsman, and T. M. Klapwijk, Phys. Rev. Appl. **9**, 064037 (2018).
[5] D. Wickramaratne, S. Khmelevskyi, D. F. Agterberg, and I. Mazin, Physical Review X **10**, 041003 (2020), publisher: American Physical Society.
[6] G. G. Taylor, A. B. Walter, B. Korzh, B. Bumble, S. R. Patel, J. P. Allmaras, A. D. Beyer, R. O'Brient, M. D. Shaw, and E. E. Wollman, Optica **10**, 1672 (2023), publisher: Optica Publishing Group.
[7] A. Semenov, A. Engel, H.-W. Hübers, K. Il'in, and M. Siegel, The European Physical Journal B - Condensed Matter and Complex Systems **47**, 495–501 (2005).
[8] A. Anikin, R. D. Schaller, G. P. Wiederrecht, E. R. Margine, I. I. Mazin, and G. Karapetrov, Physical Review B **102**, 205139 (2020), publisher: American Physical Society.
[9] X. Zhang, A. E. Lita, M. Sidorova, V. B. Verma, Q. Wang, S. W. Nam, A. Semenov, and A. Schilling, Physical Review B **97**, 174502 (2018), publisher: American Physical Society.
[10] M. V. Sidorova, A. G. Kozorezov, A. V. Semenov, Y. P. Korneeva, M. Y. Mikhailov, A. Y. Devizenko, A. A. Korneev, G. M. Chulkova, and G. N. Goltsman, Physical Review B **97**, 184512 (2018), publisher: American Physical Society.
[11] K. S. Il'in, M. Lindgren, M. Currie, A. D. Semenov, G. N. Gol'tsman, R. Sobolewski, S. I. Cherednichenko, and E. M. Gershenzon, Applied Physics Letters **76**, 2752 (2000).
[12] Y. Cao, A. Mishchenko, G. L. Yu, E. Khestanova, A. P. Rooney, E. Prestat, A. V. Kretinin, P. Blake, M. B. Shalom, C. Woods, J. Chapman, G. Balakrishnan, I. V. Grigorieva, K. S. Novoselov, B. A. Piot, M. Potemski, K. Watanabe, T. Taniguchi, S. J. Haigh, A. K. Geim, and R. V. Gorbachev, Nano Letters **15**, 4914 (2015), publisher: American Chemical Society.
[13] X. Xi, Z. Wang, W. Zhao, J.-H. Park, K. T. Law, H. Berger, L. Forró, J. Shan, and K. F. Mak, Nature Physics **12**, 139 (2016).
[14] G. Di Battista, K. C. Fong, A. Díez-Carlón, K. Watanabe, T. Taniguchi, and D. K. Efetov, Science Advances **10**, eadp3725 (2024), publisher: American Association for the Advancement of Science.
