# Supplementary material for: Single-photon detection in few-layer NbSe2 superconducting nanowires
Source: Nat Commun. 2026 Jul 29;17:7586. doi: 10.1038/s41467-026-75646-w (PMC13421474; doi:10.1038/s41467-026-75646-w)
Supplement: Supplementary file 2 — Transparent Peer Review file [file 41467_2026_75646_MOESM2_ESM.pdf]

nature portfolio

Peer Review File

Single-Photon Detection in Few-Layer NbSe2 Superconducting Nanowires

Corresponding Author: Dr Matteo Barbone

This file contains all reviewer reports in order by version, followed by all author rebuttals in order by version.

Version 0:

Reviewer comments:

Reviewer #1

(Remarks to the Author)
This work presents the experimental results of the fabrication and characterization of $NbSe_2$-based SNSPDs. Although these detectors have potential applications, some aspects should be clarified, along with questions regarding the experiment and fabrication. My comments and questions are listed below:

1) The prototype device in the form of a single wire seems impractical. Why did the authors not use a standard meander-shaped structure with higher optical coupling? Are there any issues or limitations in making larger area devices out of flakes?
2) The device response suffers from contact resistance, as can be seen clearly in Fig. 1d. The detection of lower energy photons reduces the voltage pulse amplitude, which can be obscured due to contact resistance at some point. Could the authors comment on this?
3) How was the depairing current calculated? Did you apply the Kupriyanov-Luckechev correction to the calculation? Calculating the depairing current is crucial for obtaining the correct cut-off wavelength value. Incorrect values lead to incorrect calculations of the detector potential.
4) The diffusive hot-spot model is valid for a deterministic scenario, yet the detector did not show saturation even at 780 nm. How can the $NbSe_2$ detector be made to saturate?
5) I suggest adding a zoomed-in SEM of the nanowire to see the actual quality of the edges.
6) What would be the strategy to minimize latching in $NbSe_2$ detectors? Given the limited flake area and low sheet resistance, overcoming the latching issue in $NbSe_2$ SNSPDs is quite hard.
7) I imagine that a thin flake suspended on two thick contact pads might have various effects on the detector's performance. Can the authors comment on this?
8) Does the hBN layer change the optical absorption positively or negatively?
9) How sensitive is the detector to charging or static electricity?
10) What is the device's detection efficiency?

Reviewer #2

(Remarks to the Author)
In the manuscript titled "Single-Photon Detection in Few-Layer $NbSe_2$ Superconducting Nanowires," the authors present single-photon detection using a prototype two-dimensional superconductor, $NbSe_2$, with potential applications in integrated quantum photonic circuits and quantum devices. They further claim that $NbSe_2$-based SNSPDs can extend single-photon sensitivity into the terahertz (THz) regime. However, the claim seems insufficiently substantiated, since the experimental data do not align with the theoretical predictions presented in the manuscript.
In Figure 3d, single-photon detection is observed at 1550 nm for bias currents exceeding $I/I_{sw} \approx 0.88$. This suggests that the λ_c for a 7-layer $NbSe_2$ SNSPD with a 100 nm width and $I_{bias}/I_{sw} = 0.88$ is 1.55 μm. However, in Figure 4c, the calculated λ_c for a device of similar geometry and $I_{sw}/I_{dep} = 0.5$ is about 50 μm. This discrepancy indicates that the theoretical model may significantly overestimate the cutoff wavelength, particularly when I_{sw} and I_{dep} are close. Moreover, the count rate curves in Figures 3a and 3b do not exhibit saturation behavior. A detector with a long λ_c would typically show a broad saturation plateau at the wavelength far below λ_c, as observed in WSi-based SNSPDs. The absence of such saturation further suggests that the λ_c of the present SNSPD is not in the long wavelength region.

Reviewer #3

(Remarks to the Author)
The manuscript titled "Single-photon detection in few-layer NbSe2 superconducting nanowires" presents results showing single-photon sensitivity in superconducting nanowires patterned into ultra-thin (~ 2 nm), large single-crystal grain size (multi-micron-scale), extreme uniform thickness "flakes" of NbSe2 material that were "dry-transferred" onto a standard Si/SiO2 substrate with pre-deposited gold pads. There was also a capping "flake" of hexagonal Boron Nitride to prevent oxidation, which was transferred in a similar manner. The authors show a sharp superconducting transistion and IV curves to demonstrate film quality, and trace count rate versus attenuated laser power for a range of photon fluxes and current biases to illustrate single-photon sensitivity at wavelengths of 780 nm and 1550 nm. Finally, they present some theory to make the case that this platform could in principle be extended to much longer wavelengths, well beyond mid-IR and into the THz regime.

The paper is well presented and will prove to be an important contribution to the field. I would like the authors to address some minor points and suggestions that follow. But in general, I would recommend its publication in Nature Communications.

• Abstract: Please replace "we top-down shape" with "we pattern".

• Line 53 & 79: What do you mean by "atomic thickness"? Regular SNSPD materials also boast similar thickness values to yours. Did you perhaps mean "atomic-scale roughness"?

• Line 58: I had the same question with the term "sharp interfaces."

• Figure 2: Please show the series resistor used with the voltage bias, and indicate the values of the series and shunt resistors. Do you have a means of fitting any of the timescales in the detection voltage pulse? I see two decays. Knowledge of the device inductance, line impedance,
and the bias tee inductance (and shunt resistance) should be enough for both. Granted, estimating the plateau hang time requires a lot more parameters.

• Line 217: This statement is incorrect. Propagation delay will simply shift the whole jitter histogram. Reference [4] minimized the geometric jitter by keeping the photo-sensitive area localized to a short strip.

• Line 224: This is the first appearance of Isw. Please indicate what the switching current is.

• Line 238: What do you mean by "increasing powers,"? Do you mean increasing hotspot temperature? Or increasing energy dumped into the hotspot? First-time readers might confuse this with optical power.

• Figure 3: Please replace all count rate units from kHz to kcps. Were all the error bars smaller than the marker size?

• Line 266: This section and Figure 4 is full theory, and doesn't fit well with the rest of the paper. Do you have an estimate of the depairing current from your magnetic field data from a similar device from supplemental reference [5]? Are your measured results within the ranges shown in Fig. 4(c)? It would be good to show "this work" markers somewhere there if possible.

• Line 308: Please check with the journal editors if they allow you to make future research promises. Most do not.

• Line 574: Update this arXiv reference to ACS Photonics.

• Reduce font size in both the supplemental tables.

• General: Since you didn't see saturated internal efficiency, the how dark count rate is a bit irrelevant. The timing jitter also suffered due to the same issue. However, at a Tc of 6 K, you still have a lot of room to go for even thinner flakes. Even with a Tc of 3 K or 2 K, the
quasi-particle density at 1 K should be low enough for clean signals. It would have also allowed for wider wires. Can you comment on what prevented this from being explored? Table II. in the supplemental shows a couple of flakes with fewer layers in the "This work" column.

Version 1:

Reviewer comments:

Reviewer #1

(Remarks to the Author)
2) My question was about the potential to detect mid-infrared photons, which the authors refer to in Fig. 4. As can be seen in the literature on WSi-based mid-infrared detectors, the output voltage pulse amplitude is quite low. If the detector has

additional contact resistance, reading out the pulse might be challenging.

Reviewer #2

(Remarks to the Author)
The authors explain that their discussion of the potential for THz single-photon detection in $NbSe_2$ SNSPDs is based on calculated performance achievable with a fully optimized device, rather than extrapolation from the present experimental results. Since the theoretical cut-off wavelength λc is inversely proportional to the nanowire cross-sectional area (eq.(1)), it is clear that λc extends to the millimeter range when the thickness of the nanowire is reduced. The equation is applicable not only to NbSe2 but also to other superconductors such as NbN and WSi, and is not a property unique to $NbSe_2$ SNSPDs. Compared to reference 38, the $NbSe_2$ SNSPDs fabricated in that paper showed a linear single- photon response at 1100 nm in the range of only one order of count rates due to the large dark count rate. In contrast, the present manuscript reports a single-photon detection at 1550 nm in the range of three orders, demonstrating a clear improvement in device performance.

Reviewer #3

(Remarks to the Author)
The authors have satisfactorily addressed all of my concerns from the previous round.
I can recommend this manuscript for publication.

Open Access This Peer Review File is licensed under a Creative Commons Attribution 4.0 International License, which permits use, sharing, adaptation, distribution and reproduction in any medium or format, as long as you give appropriate credit to the original author(s) and the source, provide a link to the Creative Commons license, and indicate if changes were made.
In cases where reviewers are anonymous, credit should be given to 'Anonymous Referee' and the source.
The images or other third party material in this Peer Review File are included in the article's Creative Commons license, unless indicated otherwise in a credit line to the material. If material is not included in the article's Creative Commons license and your intended use is not permitted by statutory regulation or exceeds the permitted use, you will need to obtain permission directly from the copyright holder.
To view a copy of this license, visit https://creativecommons.org/licenses/by/4.0/

Reviewer #1 (Remarks to the Author):

This work presents the experimental results of the fabrication and characterization of $NbSe_2$-based SNSPDs. Although these detectors have potential applications, some aspects should be clarified, along with questions regarding the experiment and fabrication. My comments and questions are listed below:

We thank Reviewer #1 for appreciating the potential of our platform as well as the detailed questions concerning our work and methods. The latter have been addressed point-by-point below and in the manuscript.

1) The prototype device in the form of a single wire seems impractical. Why did the authors not use a standard meander-shaped structure with higher optical coupling? Are there any issues or limitations in making larger area devices out of flakes?

The reviewer indeed raises a point worth clarifying. The main goal of the work was to realize a proof-of-principle device showing single-photon detection with conventional 2D superconductors. Thus, we chose the simplest device geometry to achieve such a goal. Fabricating a standard meander-shaped structure would have introduced additional technical hurdles, such as requiring a significantly increased heterostructure area, which is non-straightforward with exfoliated materials; additional attention to material homogeneity, defects, and environmental degradation, as well as extra optimization to tailor a more complex nanofabrication process to the novel material platform. These are obviously all steps that will be required in future devices, but go beyond the scope of the current work.

However, to clarify this aspect in the main text, we have added the following sentence in the Device Fabrication section: *"For our proof-of-concept devices, we chose the simplest geometry providing functionality without straining fabrication overhead."*

2) The device response suffers from contact resistance, as can be seen clearly in Fig. 1d. The detection of lower energy photons reduces the voltage pulse amplitude, which can be obscured due to contact resistance at some point. Could the authors comment on this?

Indeed, the contact resistance is non-negligible and can limit the single-photon detection operation. This could play a role in reducing the effective cutoff wavelength and in reaching the saturation plateau, as discussed also in the following questions. Despite

the contact resistance, though, the device showed output signals that were above the noise level and could be amplified by the read-out circuit. Also, there is indeed a difference in the output pulse depending on the incoming photon energy, but this aspect is secondary to the sensitivity of the device. Once the hotspot is created and superconductivity is broken, the pulses at different wavelengths will have a similar shape. Contact resistance affects the rising time, but it doesn't become a limiting factor to detect single photons at the wavelengths we investigated in this work.

To clarify this point, we have added a sentence in the Conclusions: *"… and render the contact resistance a negligible contribution to the detection pulse generation."*

3) How was the depairing current calculated? Did you apply the Kupriyanov-Luckechev correction to the calculation? Calculating the depairing current is crucial for obtaining the correct cut-off wavelength value. Incorrect values lead to incorrect calculations of the detector potential.

We thank the reviewer for emphasizing this point.

For the plots in Fig. 4, we did not use the calculated depairing current. We reckoned that using calculated I_{dep} would not have provided helpful information on the long-term potential of $NbSe_2$ because it would have benchmarked an infant platform against mature ones.

Thus, to calculate the potential cutoff wavelengths we considered the ratio between switching and depairing current as the free variable, and we chose several realistic values based on results from the literature that reflect state-of-the-art devices and foreseeable achievements for $NbSe_2$.

To add a comparison between calculations and real device, in the revised version we also included the cutoff wavelength of $NbSe_2$ extracted from a device reported in this work. To calculate the depairing current in this specific instance, we used the Bardeen model[1,2], which is a widely used framework in SNSPD literature:

$$I_{dep}^{Bardeen} = \frac{0.74 w \Delta(0)^{\frac{3}{2}}}{R_{sq}\sqrt{\hbar D}}$$

with $R_{sq} = \frac{1}{2e^2 D d N(0)}$ the sheet resistance, w and d nanowire width and thickness respectively, $\Delta(0)$ superconducting gap at 0 K, D electron diffusion coefficient, and N(0) density of states.

[1] https://journals.aps.org/rmp/pdf/10.1103/RevModPhys.34.667
[2] https://journals.aps.org/prapplied/pdf/10.1103/PhysRevApplied.9.064037

The Kupriyanov-Lukichev correction in the 0K approximation would only introduce a small difference[3,4]. This is due to the different prefactor. A more precise calculation of the depairing current would require a (numerical) treatment of the temperature dependence of the superconducting energy gap, which would require a substantially more detailed analysis and is therefore beyond the scope of the present work. To account for finite Temperature, we multiply $I_{dep}^{Bardeen}$by the following conventional interpolation T*dep* away from T_c, as is our case:

$$T_{dep} \sim \left[1 - \left(\frac{T}{T_C}\right)^2\right]^{\frac{3}{2}}$$

With Tc the superconducting transition temperature.

In the main text, Fig. 4c, we added the the calculated cutoff wavelength for the current device plus the following related text:

"The cross in Fig. 4c indicates the calculated cutoff for the device measured in Fig. 3, and lies well below the theoretically achievable cutoff, reflecting the technological immaturity of the platform and indicating significant room for further optimization [...] All material parameters and details of the calculations are included in the Suplementary Information."

In the Supplementary material, we added the formula and a table with the parameters used to calculate the depairing current of our device. We also added the following text:

"To calculate the data point in Figure 4, we used the parameters presented in Table SII, which are measurement parameters (T), extracted from our device (w, Δ, $R_{\square}$, T_c) or taken from literature (D)[5]. The depairing current is calculated using the following formula [Bardeen, Rev Mod Phys 34, 667, 1962]: " [formula and description follow]

4) The diffusive hot-spot model is valid for a deterministic scenario, yet the detector did not show saturation even at 780 nm. How can the $NbSe_2$ detector be made to saturate?

To achieve saturation, the device geometry could be modified, decreasing its cross-section – reducing the thickness or the width. This way, the ratio between geometry and hot-spot size will be more favourable. Moreover, reducing the critical temperature would also increase the sensitivity. Finally, another way to achieve saturation would require an optimized fabrication, allowing for an increase in the operational current compared to the

[3] https://journals.aps.org/prb/abstract/10.1103/PhysRevB.86.174521
[4] https://journals.aps.org/prb/abstract/10.1103/PhysRevB.100.054520

depairing current. In our device, saturation is further limited by latching, which, once removed, would allow for higher biasing currents.

To address this point, we added a sentence in the discussion of Fig.3: *"Modifying the geometry of the device to reduce its cross-section and enhancing its transport properties to increase the bias current closer to the depairing current would overcome latching and therefore enable the device to reach saturation."*

And in the Conclusions: *"fabrication, allowing the fabrication of larger suitable active areas and the realization of geometries and architectures -such as meander-shape geometry or on-chip inductors and resistors- to minimize latching."*

5) I suggest adding a zoomed-in SEM of the nanowire to see the actual quality of the edges.

We thank the reviewer for the insightful suggestion. We include here and in the supplementary information a SEM image of the nanowire showing the edge roughness. The wire has a width of around 100 nm. The images show polymeric residuals, highlighting the potential for quality and device fabrication improvements.

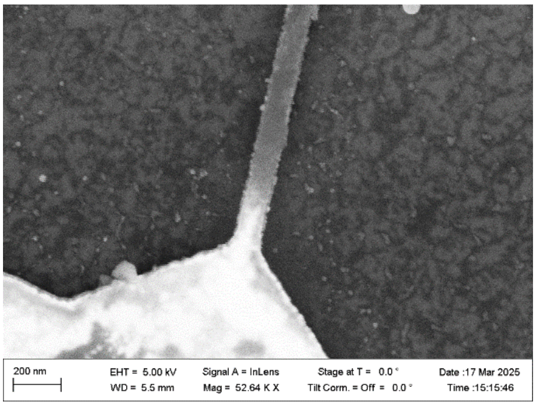

6) What would be the strategy to minimize latching in $NbSe_2$ detectors? Given the limited flake area and low sheet resistance, overcoming the latching issue in $NbSe_2$ SNSPDs is quite hard.

To minimize latching in $NbSe_2$ detectors, we would design a device with a higher inductance. This would be possible by increasing the nanowire length – meander shape – or by adding a superconducting inductor on chip. Moreover, latching can be reduced by adding a shunt/parallel resistor closer to the nanowire.

Similarly to above, in the main text, we added additional information concerning latching, specifically in the discussion of Figure 3:

"Modifying the geometry of the device to reduce its cross-section and enhancing its transport properties to increase the bias current closer to the depairing current would overcome latching and therefore enable the device to reach saturation."

And in the Conclusions:

"fabrication, including geometries and architectures -such as meander-shape geometry or on-chip inductors and resistors- to minimize latching and maximise photon absorption at operational wavelength. Moreover, an increased wire-length would increase the device inductance and reduce the contact resistance to a negligible contribution to the detection pulse generation."

7) I imagine that a thin flake suspended on two thick contact pads might have various effects on the detector's performance. Can the authors comment on this?

In the case of a suspended flake between thick contact pads, the thermal conduction of the device would change. The heat deposited from the photon would be dissipated only along the device plane, given the limited thermal capacity of ~nm thin hBN as an encapsulating layer. This would enhance the device sensitivity due to the confined channel[5].
Nevertheless, we point out that our devices do not consist of a free-standing membrane, but the wire is in contact with the substrate. The area closer to the contact pads consists of a wider structure to ensure a more stable and robust connection.

To clarify this point, we have added a sentence in the description of Figure 1: *"... and the oxidized silicon substrate... "*

And in the Methods section: *"The nanowire structure around the contact pads was designed to ensure a robust contact between the nanowire and the contact pads."*

8) Does the hBN layer change the optical absorption positively or negatively?

The central role of hBN in this work is to provide passivation to $NbSe_2$. However, given the refractive indexes of the materials, adjusting the thickness of the hBN layer (and of the whole hBN/$NbSe_2$/SiO_2 stack, to which also other layers can be added), creates a half-cavity enhancing (or suppressing) photon absorption at specific wavelengths, see e.g. [6]. In our work, we started from 130 nm SiO_2 on Si wafers, and we designed heterostructures by selecting hBN crystals with a thickness that may provide good absorption at both relevant laser energies. However, since 780 and 1550 nm sit very far apart, inevitably, the

[5] https://pubs.aip.org/aip/apl/article-abstract/123/8/082602/2907714/
[6] https://pubs.aip.org/aip/apl/article/91/6/063124/1023191

same device is not optimized for each wavelength. Nevertheless, the sensitivity is independent of the absorption efficiency, therefore, not limiting the scope of this work.

9) How sensitive is the detector to charging or static electricity?

The device is sensitive to electric discharge, hence care is necessary when preparing the sample for operation (bonding, room temperature characterization, ...), but we did not find it to be an issue for operation and measurement under controlled conditions.

10) What is the device's detection efficiency?

We thank the reviewer for raising this point, which is relevant in the SNSPD community. Determining an absolute detection efficiency is a good metric to compare devices with state-of-the-art systems.

The primary goal of this work was to demonstrate proof-of-principle operation of $NbSe_2$ for single photon detection, as demonstrated by the photon statistic presented in Figure 3. In fact, we did also calculate the detection efficiency, but without it being our primary focus. Amongst other points, also touched on in previous questions, we did not fabricate a meander structure, we did not incorporate optimized resonators to maximise optical absorption, and we did not optimize the optical configuration to our specific devices. These aspects require separate dedicated efforts and would be the subject of future work. Thus, the calculated device efficiency should be taken as a very preliminary approximation of the bottom end of the achievable efficiency range.

With these aspects in mind, to answer fully, we include the following Figure for review purposes showing the calculated detector efficiency as a function of bias voltage and number of incident photons per second:

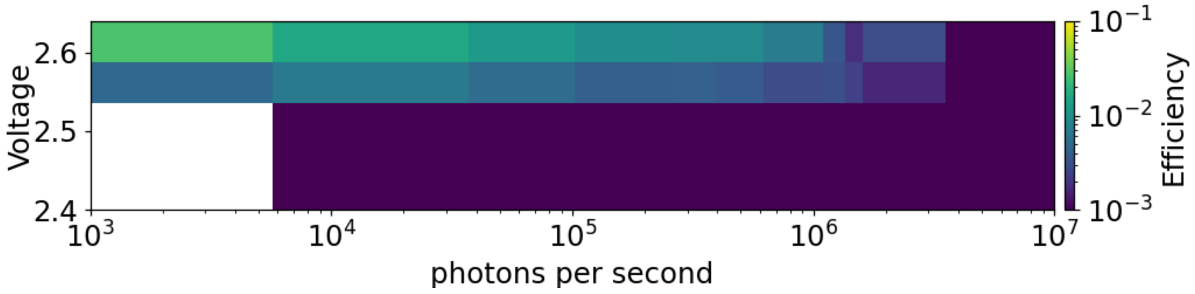

Figure 2: Colour map showing the detector efficiency (counts per second/incident photons) as function of incident photons and bias Voltage.

As the Figure shows, the efficiency reaches a maximum < 3%.Given the context of the results, we reckon that they hold little interest for the community and may rather be more misleading than helpful. Indeed, the absolute efficiency is low compared to mature platforms, as expected for a new, unoptimized material, but the non-specialist or skimming reader may still be brought to compare such values against optimized, technologically mature SNSPD platforms. For such reasons, and in line with the approach of analogous proof-of-concept studies[7], we prefer to omit such information in the manuscript.

[7] https://www.nature.com/articles/s41565-023-01325-2

Reviewer #2 (Remarks to the Author):

In the manuscript titled "Single-Photon Detection in Few-Layer $NbSe_2$ Superconducting Nanowires," the authors present single-photon detection using a prototype two-dimensional superconductor, $NbSe_2$, with potential applications in integrated quantum photonic circuits and quantum devices.

We thank the Reviewer for the attention paid to our work and for highlighting its longer-term prospects.

They further claim that $NbSe_2$-based SNSPDs can extend single-photon sensitivity into the terahertz (THz) regime. However, the claim seems insufficiently substantiated, since the experimental data do not align with the theoretical predictions presented in the manuscript.

We thank the Reviewer for raising this concern, which allowed us to revise and clarify our analysis of the potential for long-wavelength detection, as detailed below.

In Figure 3d, single-photon detection is observed at 1550 nm for bias currents exceeding I/Isw ≈ 0.88. This suggests that the λc for a 7-layer $NbSe_2$ SNSPD with a 100 nm width and Ibias/Isw = 0.88 is 1.55 μm. However, in Figure 4c, the calculated λc for a device of similar geometry and Isw/Idep = 0.5 is about 50 μm. This discrepancy indicates that the theoretical model may significantly overestimate the cutoff wavelength, particularly when Isw and Idep are close. Moreover, the count rate curves in Figures 3a and 3b do not exhibit saturation behavior. A detector with a long λc would typically show a broad saturation plateau at the wavelength far below λc, as observed in WSi-based SNSPDs.

We understand the concerns raised by the Reviewer towards the discrepancy between the theoretical calculations and our measured data, but we believe they stem from a misunderstanding of the nature and purpose of our theoretical projections.

We have clarified the scope and validity of our analysis below and in the manuscript.

The absence of such saturation further suggests that the λc of the ***present*** (boldened by us) *SNSPD is not in the long wavelength region.*

We feel that this sentence encapsulates the misunderstandings created by the analysis in Fig. 4, and we apologize for the confusion.

We agree that our current device will have a lower cutoff wavelength than 50 μm.

First, in the calculation of λc for all platforms, we assumed a conversion efficiency ζ =1. This was done to level the playing field between mature, developing, and novel platforms. Obviously, the real value of ζ will be lower than 1.

Second, upon further inspection, we noticed that the original $NbSe_2$ and the WSi relaxation times (based on the electron-phonon scattering time) were not taken at the operational temperature and were underestimated due to the strong temperature-dependence of electron-phonon interaction. In the revised calculations, the expected cutoff wavelengths are now lower than our previous estimates due to longer relaxation times.

Third, most importantly our primary concern was not to extrapolate the performance of the $NbSe_2$ devices realized in this work, but to preliminarily gauge the potential of $NbSe_2$ as a platform for long-wavelength operations in future optimized configurations, beyond the limited experimental results of our proof-of-principle study.

To this end, we start by considering that the cutoff wavelength formula employs both *materials* and *device* parameters. For NbN- and WSi-based SNSPDs, realistic and mature experimental values are available for both materials and device parameters because of the advancement of such technological platforms. For $NbSe_2$, reliable intrinsic materials parameters are available, since they rest on a solid base of fundamental studies; in contrast, device parameters are not, because the manuscript under consideration reports the first proof-of-concept $NbSe_2$ SNSPD. Thus, the current device parameters are extremely immature and very far from what an optimized device will realistically yield.

However, optimizing devices will require a significant amount of future effort that we feel would be more motivated by offering the community a clear outlook on the potential benefits of $NbSe_2$.

Thus, we reckoned that benchmarking the perspectives of $NbSe_2$ by using mature device parameters for WSi and NbN against embryonic ones for $NbSe_2$ would have resulted in an uneven comparison. Thus, we decided that a more useful approach would have been to calculate what $NbSe_2$-based devices may yield, *assuming* that future efforts can emulate the progress seen in mature SNSPD material platforms and achieve comparable I_{sw}/I_{dep}.

Our message is that $NbSe_2$ offers favourable materials parameters, namely a 2D nature, which potentially allows for sub nm thickness (1L being ~0.7 nm), and fast thermalization time. To improve device parameters, several technological problems will need to be overcome, including optimizing nanofabrication to increase the achievable I_{sw}/I_{dep} and

bringing it closer to materials such as NbN or WSi, where it can reach values of ~0.7[8,9], as well as overcoming latching, which further reduces the switching current and the achievable saturation plateau.

To clarify our purpose, we have edited the text as follows:

Abstract:

~~*"We use a diffusive hot-spot model to estimate a theoretical cut-off wavelength that surpasses the millimetre range."*~~

"We estimate via a diffusive hot-spot model that materials and geometric parameters would allow a theoretical cut-off wavelength in the millimetre range, assuming state-of-the-art device parameters."

Main text:

"Assuming that future research developments in $NbSe_2$ SNSPD can match the significant improvement of device parameters reached in technologically mature systems, we explore the advantages offered by materials parameters of $NbSe_2$ for long wavelength detection."

Removing the previous sentence:

"~~Atomically thin conventional layered superconductors are especially intriguing for detecting low-energy excitations because of single crystal nature and atomically precise thickness.~~"

Moreover, to further clarify this point, we explicitly mention that I_{sw}/I_{dep} is used as a free parameter in the calculation as well as the assumed conversion efficiency by modifying the following sentence:

"Since the quasi-particle multiplication efficiency as well as the current component are strongly affected by technological maturity, underestimating the potential of newer material systems, we calculate λc assuming $\zeta = 1$ and using I_{bias}/I_{dep} as a free parameter in a realistic range achievable by state-of-the-art devices [Parzuchowski et al., arXiv:2601.15971; Frasca et al., Physical Review B 100, 054520 (2019)]

However, for completeness, in Fig 4c we have calculated the expected cutoff wavelength for our current devices based on the measured Isw/Idep ratio (<0.1), indicated by a cross. The new version of the Supplementary now includes details on the depairing current calculation and on all parameters used. The theoretical cutoff of the current device is ~9

[8] https://arxiv.org/pdf/2601.15971
[9] https://journals.aps.org/prb/pdf/10.1103/PhysRevB.100.054520

um, orders of magnitude lower than what should be achievable with optimized devices, reflecting a the limitation from a 7-layer thick film and low I_{sw}/I_{dep}. Again, this calculation still likely overestimates the cutoff of the device due to experimental imperfections, analogous to the other platforms.

Reviewer #3 (Remarks to the Author):

The manuscript titled "Single-photon detection in few-layer NbSe2 superconducting nanowires" presents results showing single-photon sensitivity in superconducting nanowires patterned into ultra-thin (~ 2 nm), large single-crystal grain size (multi-micron-scale), extreme uniform thickness "flakes" of NbSe2 material that were "dry-transferred" onto a standard Si/SiO2 substrate with pre-deposited gold pads. There was also a capping "flake" of hexagonal Boron Nitride to prevent oxidation, which was transferred in a similar manner. The authors show a sharp superconducting transistion and IV curves to demonstrate film quality, and trace count rate versus attenuated laser power for a range of photon fluxes and current biases to illustrate single-photon sensitivity at wavelengths of 780 nm and 1550 nm. Finally, they present some theory to make the case that this platform could in principle be extended to much longer wavelengths, well beyond mid-IR and into the THz regime.

The paper is well presented and will prove to be an important contribution to the field. I would like the authors to address some minor points and suggestions that follow. But in general, I would recommend its publication in Nature Communications.

We thank Reviewer #3 for the strongly supportive assessment and for the concrete suggestions provided to improve the manuscript, both on content and readability. Below, we address each point raised.

• *Abstract: Please replace "we top-down shape" with "we pattern".*

Thanks. Text updated accordingly.

• *Line 53 & 79: What do you mean by "atomic thickness"? Regular SNSPD materials also boast similar thickness values to yours. Did you perhaps mean "atomic-scale roughness"?*

Thank you for pointing out a possible misunderstanding. Concerning the atomic thickness attribute of 2D materials, we refer to the capability to mechanically and deterministically select materials with specific atomic layers, compared to the standard sputtered/amorphous materials. We modified the text in the Introduction, adding the following sentence to avoid confusion: *"...controllable atomic thickness (layer-by-layer)..."* and *"...single layer..."*

• *Line 58: I had the same question with the term "sharp interfaces."*

We clarified that the sharp interfaces refer to the atomically abrupt van der Waals interfaces.

Text updated as follows: *"...between layers..."*

• *Figure 2: Please show the series resistor used with the voltage bias, and indicate the values of the series and shunt resistors. Do you have a means of fitting any of the timescales in the detection voltage pulse? I see two decays. Knowledge of the device inductance, line impedance, and the bias tee inductance (and shunt resistance) should be enough for both. Granted, estimating the plateau hang time requires a lot more parameters.*

During the experiment, we did not utilize a series resistor, but utilized only the intrinsic resistance of the setup (wiring resistance, contact resistance, ...); no series resistor is therefore included in the figure. The value of 50 Ω of the parallel resistor (shunt) is included in the caption of Figure 2: *"...R = 50 Ω..."*

We appreciate the feedback regarding the timescale estimation for the voltage pulse. We investigate and discuss the decay behaviour of the pulse in the Supplementary Information. Here, we calculate the kinetic inductance of the device starting from the device properties (resistance and critical temperature). This is then directly connected to the recovery time by the formula $\tau \approx \frac{L_k}{Z_0}$, where Z_0 is the circuit impedance (50 Ω), resulting in a recovery time of ~ 6.38 ps. The resulting value is considerably smaller compared to standard devices produced from other materials and different geometries, which can be explained by the different material properties and the geometry of the fabricated device. As described in the Supplementary Information, this causes the device to latch, therefore generating a pulse shape where the decay shape is governed by latching rather than the standard response of the superconducting detector.

The temporal dynamics of $NbSe_2$ are indeed an open field of investigation, which could once again show great promise compared to standard materials used for single-photon detection. Nevertheless, the scope of the work was focused on the optical response of the device and its possible application to lower-energy photons, demonstrating its feasibility, leaving additional studies for future endeavors.

• *Line 217: This statement is incorrect. Propagation delay will simply shift the whole jitter histogram. Reference [4] minimized the geometric jitter by keeping the photo-sensitive area localized to a short strip.*

The Reviewer is indeed correct, thanks for pointing this out. We modified the text with further details as expressed in the cited paper, adding the following sentence: *"...simultaneously minimize the longitudinal geometric and intrinsic jitter."*

• *Line 224: This is the first appearance of Isw. Please indicate what the switching current is.*

The switching current was previously introduced at line 143. Nevertheless, for ease of understanding and readability, we added a clarification in line 224: *"...normalized to the switching current..."*

• *Line 238: What do you mean by "increasing powers,"? Do you mean increasing hotspot temperature? Or increasing energy dumped into the hotspot? First-time readers might confuse this with optical power.*

To increase clarity on this point, we updated the text to clearly state the relation to the increasing photon flux: *"...count rates..."*

• *Figure 3: Please replace all count rate units from kHz to kcps. Were all the error bars smaller than the marker size?*

The plots have been updated towards different units from kHz to kcps.

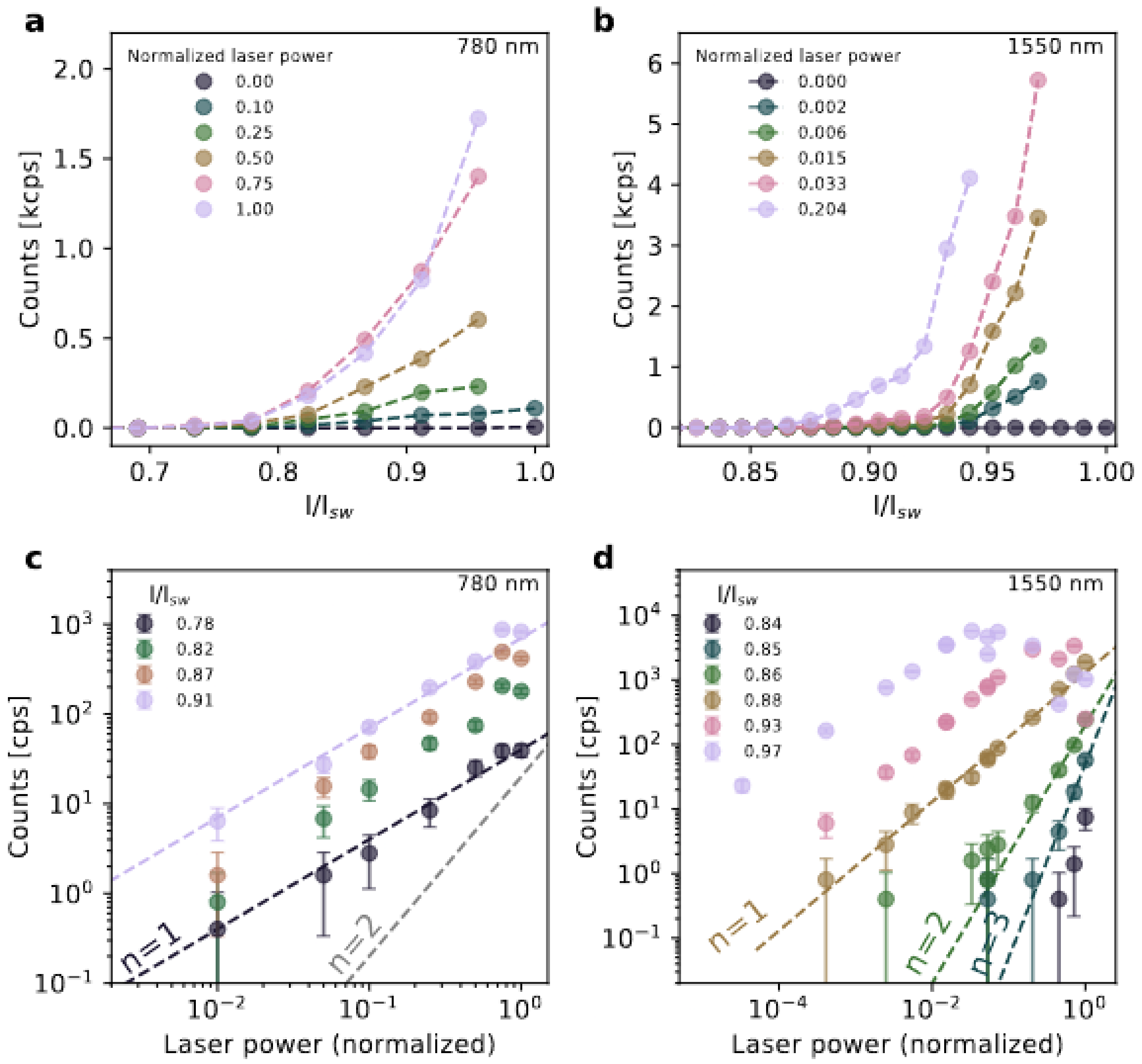

The uncertainty in the data is shot noise-limited and follows a relative uncertainty of $\frac{1}{\sqrt{N}}$, an absolute uncertainty of $\sqrt{N}$. Therefore, in the current sweeps plots, which are linear, the error bars are smaller than the marker size, while in the logarithmic plots, the error bars are visible for low count rates. We included in the caption of the figure the following text to address this point: *"... (error bars smaller than the marker size) ..., where the uncertainty is shot noise limited."*

• *Line 266: This section and Figure 4 is full theory, and doesn't fit well with the rest of the paper. Do you have an estimate of the depairing current from your magnetic field data from a similar device from supplemental reference [5]? Are your measured results within the ranges shown in Fig. 4(c)? It would be good to show "this work" markers somewhere there if possible.*

Thank you for the comment raised. The scope of the following section concerns the forecasted benefits of $NbSe_2$ as a material for single-photon detection. Several modifications to the text have been added to avoid misunderstandings or to mislead the reader. We calculated the depairing current (Supplementary Information) and found the I_{sw}/I_{dep} to be <0.1, reflecting the technological immaturity of the platform. We also modified Figure 4 as suggested, showing the position of the device presented in this work. As mentioned previously in this Response letter and now more clearly discussed in the manuscript, our device is predicted to offer a much lower cutoff wavelength (~9 µm) than the calculated scenarios, mainly due to the low I_{sw}/I_{dep}.

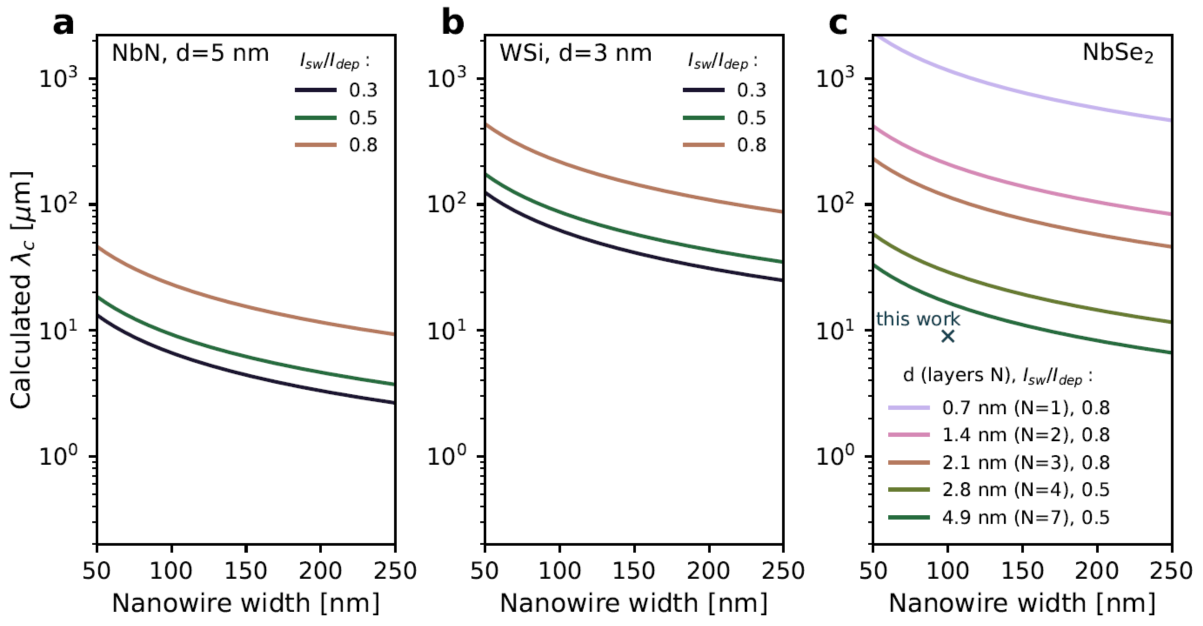

To benchmark the performance of our device, we considered material properties collected from our device or from literature, as mentioned in the Supplementary, and used the Bardeen model[10,11], which is a widely used framework in SNSPD literature, to calculate the depairing current of our device. With the resulting I_{sw}/I_{dep} ratio < 0.1 for our device, we see that the cutoff wavelength is in the order of ~25 µm. The discrepancy between theoretical value and experimental finding is common for all materials, where there is a factor of 5-7 for established material systems (NbN and WSi) and a factor of 15-20 for $NbSe_2$, in its first demonstration of single-photon sensitivity. This factor and the low I_{sw}/I_{dep} ratio are common for early-stage materials and are further accentuated by the device limitations, such as latching.

We added in the Supplementary materials a table with the parameters used to calculate the depairing current of our device, and added the following text:

---

[10] https://journals.aps.org/rmp/pdf/10.1103/RevModPhys.34.667
[11] https://journals.aps.org/prapplied/pdf/10.1103/PhysRevApplied.9.064037

“To calculate the data point in Figure 4, we used the parameters presented in Table SII, which are measurement parameters (T), extracted from our device (w, Δ, $R_\square$, T_c) or taken from literature (D) [Metuh et al., ACS Photonics 12, 5912 (2025).”

Moreover, we modified the caption of Figure 4 as follows:

“The cross represents the predicted cutoff wavelength for the device presented in this work, with a $I_{bias}/I_{dep} < 0.1$.”

We added in the main text describing Figure 4 the following modifications. Text added:

“The device considered in this work, indicated by the cross and the '*This work*' label, lies below these projections, consistent with the early-stage development of the platform, and indicating significant room for further optimization. Even so, the overall increase in expected λ_c provided by $NbSe_2$ is remarkable, despite the assumptions and approximations of our model.”

Removing the previous text:

“Despite the assumptions and approximations of our model, the overall increase in expected λ_c provided by $NbSe_2$ is remarkable.”

- *Line 308: Please check with the journal editors if they allow you to make future research promises. Most do not.*

Thank you for pointing out this concern. We will confirm with the journal editors.

- *Line 574: Update this arXiv reference to ACS Photonics.*

Reference updated accordingly.

- *Reduce font size in both the supplemental tables.*

Thank you for commenting on the readability of the tables. Old and new Tables adjusted accordingly.

TABLE I. Material and device parameters used to calculate depairing current.

Parameter	Value
w [nm]	100
$\Delta = k_B T_c$ [meV]	0.516
$R_\square$ [$\Omega/\square$]	14
D [$m^2 s^{-1}$]	1×10^{-5} [2]
T [K]	1
T_c [K]	6

CUTOFF WAVELENGTH EFFICIENCY CALCULATIONS

TABLE II. Material parameters used in the diffusion-based hot-spot model.

Parameter	$NbSe_2$	WSi	NbN
N_0 [10^{47} $m^{-3} J^{-1}$]	1.3[5]	1.08[6]	1.6[7]
$\Delta = k_B T_c$ [meV]	0.43 ($T_c = 5$ K)	0.112 ($T_c = 1.3$ K)	0.43 ($T_c = 5$ K)
D [$m^2 s^{-1}$]	1×10^{-5} [2]	5.3×10^{-5} [6]	3.5×10^{-5} [7]
τ_{th} [ps]	10[8]	120[9, 10]	12[10, 11]
d [nm]	0.7-bulk	3[6]	5[7]

TABLE III. Thickness dependent critical temperature for $NbSe_2$.

Layers	Cao *et al.* [K] [12]	Xi *et al.* [K] [13]	This work [K]
1	1.95	3.1	-
2	4.5	5.4	5.1
3	5.5	5.9	5.6
4	-	6.2	-
7	6.5	6.9	6.0

• *General: Since you didn't see saturated internal efficiency, the how dark count rate is a bit irrelevant. The timing jitter also suffered due to the same issue. However, at a Tc of 6 K, you still have a lot of room to go for even thinner flakes. Even with a Tc of 3 K or 2 K, the quasi-particle density at 1 K should be low enough for clean signals. It would have also allowed for wider wires. Can you comment on what prevented this from being explored? Table II. in the supplemental shows a couple of flakes with fewer layers in the "This work" column.*

Thank you for the valid point that you raised. The main issue that we faced in operating the other devices came from the high contact resistance of the devices themselves. Changing the overall cross-section of the geometry would improve the sensitivity of the device to longer wavelengths, but with this thickness, a wider wire would further accentuate latching in the device.

Nevertheless, this work aimed to demonstrate the proof-of-principle of the device, its sensitivity to single photons, and the conceptual capabilities for long-wavelength detection. The main challenge to achieve devices with higher sensitivity lies mainly in improving the device exfoliation and the nanofabrication steps.

To address these points, additional text was added in the Conclusions outlining the necessary steps to achieve state-of-the-art performances.

Text updated as follows: *"...fabrication, including geometries and architectures -such as meander-shape geometry or on-chip inductors and resistors- to minimize latching and maximise photon absorption at operational wavelength. Moreover, an increased wire-length would increase the device inductance and reduce the contact resistance to a negligible contribution to the detection pulse generation. With these implementation, single-photon detection can be expanded to the MIR".*

REVIEWERS' COMMENTS

Reviewer #1 (Remarks to the Author):

2) My question was about the potential to detect mid-infrared photons, which the authors refer to in Fig. 4. As can be seen in the literature on WSi-based mid-infrared detectors, the output voltage pulse amplitude is quite low. If the detector has additional contact resistance, reading out the pulse might be challenging.

We thank the reviewer for the clarification concerning the connection between pulse amplitude and longer wavelength detection. Surely contact resistance will need to be consistently minimized. Although the main parameter impacting the pulse amplitude is the applied current to the device during the detection event. As described in the paper from Taylor and coauthors about the detection of 29µm photons using WSi devices, the limitation to the pulse amplitude comes from the low Switching current of the device, which is limited by the geometrical cross section and from the low critical temperature of the device needed.

$NbSe_2$ will face similar challenges from the geometrical cross-section side, but at the same time materials properties, in particular Tc, result in a higher expected switching current.

Reviewer #2 (Remarks to the Author):

The authors explain that their discussion of the potential for THz single-photon detection in $NbSe_2$ SNSPDs is based on calculated performance achievable with a fully optimized device, rather than extrapolation from the present experimental results. Since the theoretical cut-off wavelength λc is inversely proportional to the nanowire cross-sectional area (eq.(1)), it is clear that λc extends to the millimeter range when the thickness of the nanowire is reduced. The equation is applicable not only to NbSe2 but also to other superconductors such as NbN and WSi, and is not a property unique to $NbSe_2$ SNSPDs.
Compared to reference 38, the $NbSe_2$ SNSPDs fabricated in that paper showed a linear single- photon response at 1100 nm in the range of only one order of count rates due to the large dark count rate. In contrast, the present manuscript reports a single-photon detection at 1550 nm in the range of three orders, demonstrating a clear improvement in device performance.

We thank Reviewer 2 for the fruitful discussion and the clarifications that followed. This helped to improve the overall structure and readability of the paper.
Reviewer #3 (Remarks to the Author):

The authors have satisfactorily addressed all of my concerns from the previous

round.
I can recommend this manuscript for publication.

We thank Reviewer 3 for the positive feedback and the helpful insights on how to improve the presentation of the paper.
